# Herpetic Keratitis Following Treatment of Acute Antibody-Mediated Rejection in a Renal Transplantation Recipient

**DOI:** 10.7759/cureus.51711

**Published:** 2024-01-05

**Authors:** Geeta Behera, Sandip Sarkar, Jagadeeshwari Jayaseelan, Sreejith Parameswaran

**Affiliations:** 1 Ophthalmology, Jawaharlal Institute of Post-Graduate Medical Education and Research (JIPMER), Puducherry, IND; 2 Nephrology, Jawaharlal Institute of Post-Graduate Medical Education and Research (JIPMER), Puducherry, IND

**Keywords:** antiviral therapy, acute graft rejection, immunosuppression, post kidney transplant, herpetic keratitis

## Abstract

We report an incident case of herpetic keratitis in a renal transplant recipient treated for acute renal allograft rejection. A lady in her forties, a renal transplant recipient on treatment for allograft rejection, was referred with mild ocular symptoms in the right eye for two days. On evaluation, she had mild conjunctival hyperemia and extensive herpetic epithelial keratitis involving the limbal and central corneas. The patient healed without sequelae from the antivirals and lubricants. Viral keratitis in immunosuppressed patients should be suspected, even in patients with mild symptoms, as early initiation of treatment can prevent rapid stromal involvement and scarring.

## Introduction

Tremendous advances in immunology and immunosuppressive therapy have improved graft and patient survival in renal transplantation for end-stage renal disease (ESRD) [1]. However, lifelong immunosuppressive therapy predisposes these patients to numerous complications. Ocular complications can vary from posterior subcapsular cataracts and high intraocular pressure (due to long-term steroid use) to uncommon opportunistic ocular infections such as cytomegalovirus (CMV), herpes simplex, herpes zoster, cryptococcosis, mucormycosis, candidiasis, aspergillosis, and toxoplasmosis [2]. Herpetic eye disease includes keratitis, corneal ulcers, uveitis, and retinitis, characterized by large areas of retinal necrosis. Herpetic epithelial keratitis following renal transplantation has been reported in two case series published three decades ago [3,4]. We report an incident case of herpetic keratitis in a post-renal transplant recipient treated for acute renal allograft rejection.

## Case presentation

A lady in her forties was referred to us with complaints of mild redness, watering, photophobia, and slight blurring of vision in the right eye for the last two days. She had chronic kidney disease (of an undetermined cause) and had undergone deceased donor renal transplantation one and a half years prior. She received thymoglobulin at the time of transplantation, followed by immunosuppression with tacrolimus 10 mg per day, mycophenolate mofetil (MMF) 2 g per day, and prednisolone 10 mg per day. She had hypothyroidism and systemic hypertension and developed post-transplant diabetes mellitus (PTDM). Eight months after renal transplantation, she developed severe anemia and was found to have parvovirus-induced pure red cell aplasia (PRCA). The patient was successfully treated with multiple packed cell transfusions, reduction of immunosuppression (MMF stopped and tacrolimus dose reduced to 6 mg per day), and intravenous immunoglobulin (IVIg). During her hospital stay, she developed COVID-19 (diagnosed by reverse transcription-polymerase chain reaction), which was uncomplicated. She was simultaneously diagnosed with sputum-positive pulmonary tuberculosis and initiated rifampicin-free anti-tubercular therapy (isoniazid 300 mg/day, ethambutol 1200 mg/day, pyrazinamide 1550 mg/day, and levofloxacin 750 mg/day for six months). Ten days before the ophthalmology referral, she developed worsening renal allograft function, which was determined to be from acute antibody-mediated renal allograft rejection on allograft biopsy, as per the Banff criteria. She was started on plasma exchange (PLEX), IVIg, and an optimized tacrolimus dose of 6 mg per day, based on plasma trough levels.

**Figure 1:**
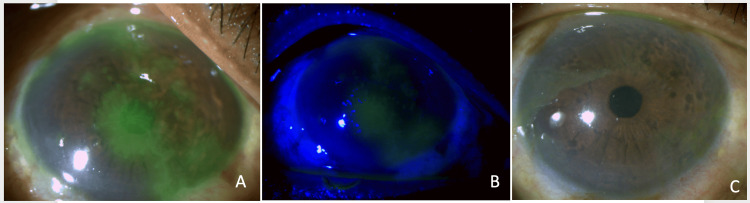
Slit-lamp photographs (A) Large epithelial dendritic pattern linear branching lesions with terminal bulbs involving the central and limbal cornea; (B) the dendritic ulcer viewed under cobalt blue filter; (C) healed cornea at four weeks.

Her best-corrected visual acuity (BCVA) was 20/32 in the right eye and 20/25 in the left eye. Intraocular pressure was normal: OD, 15 mmHg; OS, 17 mmHg. A slit-lamp examination of the right eye revealed mild circumciliary congestion and hyperemia. Multiple epithelial linear branching lesions with terminal bulbs in dendritic patterns involving almost the entire central cornea and limbal areas were noted (Figure 1A-1B). The corneal sensation was reduced in the right eye. The anterior chamber was devoid of inflammation, and pupillary reactions were brisk. Her left eye was normal. Fundus examination revealed mild, non-proliferative diabetic retinopathy in both eyes. Her history did not suggest it, and a general examination did not reveal any prior or recent-onset skin/mucosal herpetic infections. Based on the clinical findings, a diagnosis of herpetic keratitis post-immunosuppression for renal transplant rejection was made.

The patient started ganciclovir 0.15% eye ointment five times a day, carboxymethylcellulose eye drops 0.5% hourly, and moxifloxacin 0.5% eye ointment three times per day. She continued treatment for renal allograft rejection with uninterrupted IVIg and PLEX. The dendritic lesions healed within one week of antiviral therapy, and the topical antibiotic was discontinued. Topical antiviral treatment was continued for four weeks. At the last follow-up visit (three months after healing), her BCVA was 20/25 (OU), and her right cornea had completely healed without any scarring (Figure 1C).

## Discussion

Renal transplant patients require lifelong immunosuppression to prevent allograft rejection, making them susceptible to opportunistic infections. Due to systemic immunosuppression, extraocular herpes simplex and other viral infections are well documented following kidney transplantation [5]. A recent report showed that up to 63.6% of patients treated for acute rejection developed infectious complications within six months, with plasmapheresis increasing the risk of these infections [6]. PLEX and IVIg used in our patient are accepted standard treatments for acute antibody-mediated renal allograft rejection [6]. However, epithelial keratitis due to the herpes simplex virus (HSV) in renal transplant recipients is extremely rare [3,4]. Two series have reported it following renal transplantation three decades prior. These patients received triple immunosuppressive therapy with prednisone, azathioprine, and cyclosporine [3,4]. Our patient developed herpetic epithelial keratitis following systemic immunosuppression prescribed for acute graft rejection without any prior history of ocular disease. Before the ophthalmology referral, she had mild ocular symptoms with a diminution of vision for two days. We did not consider Varicella Zoster keratitis as there were no skin lesions in the trigeminal distribution, and the keratitis was not pseudo-dendritic in appearance. We did not perform molecular diagnostics, as clinically, it was herpetic dendritic keratitis (Figure 1A-1B) that responded immediately to antiviral therapy. Also, polymerase chain reaction (PCR) is positive in 77% of cases of acute epithelial keratitis and does not usually guide treatment in such clear cases of viral keratitis [7].

Epithelial keratitis is the most common ocular presentation of herpetic eye disease and may be primary, latent, or recurrent. After primary infection, the virus can stay dormant in the trigeminal ganglia. Neuronal latency has been found to increase with age, reaching 100% in persons aged greater than 60 years [8]. Ocular reactivation of HSV in stress and immunocompromised states, including immunosuppressive therapy following transplantation, etc., is well documented [3,4,9,10]. HSV epithelial keratitis following systemic immunosuppression therapy for acute renal graft rejection is distinctly rare and probably underreported [3]. Two previous series reported atypical involvement with minimum symptoms and conjunctival hyperemia, dendritic ulcers near the limbus and multiple dendrites, bilateral involvement, rapid involvement of the stroma, and the requirement for a longer duration of antiviral therapy [3,4]. Our patient, too, had mild symptoms and minimal conjunctival hyperemia but extensive limbal and central corneal lesions. It may be noted that normal patients who develop acute viral keratitis are usually very symptomatic and complain of severe pain, red eyes, and copious watering. These symptoms may be relatively blunted owing to systemic immunosuppression in post-transplant patients. Among the four patients reported by Howcroft and Breslin, two patients detected early (three to four days) with viral keratitis healed well upon initiating therapy [3]. The other two patients, referred after a week, had associated herpes labialis and recovered after a protracted course with corneal scarring affecting vision. It should be noted that 0.5% idoxuridine ointment was the only antiviral treatment available at the time. Kremer et al. reported prior skin involvement in three of five patients and healing after three to four weeks of antiviral therapy (acyclovir ointment) with superficial scarring due to nummular keratitis [4]. However, our patient did not report any prior herpetic eye or skin/mucosal disease. She was referred on the third day after developing ocular symptoms, which healed without sequelae within a week of antiviral therapy with topical ganciclovir. Ganciclovir is reported to be as effective as acyclovir in the management of herpetic epithelial keratitis [11].

Early referral and identification aided in initiating effective therapy, contributing to a quick resolution without sequelae. Although herpetic epithelial keratitis is very rare following renal or solid organ transplantation, it can progress rapidly to involve the corneal stroma if not identified due to systemic immunosuppression. Early detection may also be missed, as the patient may not be very symptomatic and the eye may appear white despite keratitis [4]. Previous cases also suggest that herpetic skin or oral mucosa involvement may predispose these patients to viral keratitis. Hence, adequate prevention through early and sustained topical/oral antiviral therapy and appropriate hygiene to prevent cross-contamination may be considered. Fortunately, owing to advances in therapy, the treatment of viral keratitis is successful in most cases. However, immunosuppressed patients are particularly at risk of subsequent corneal scarring, resulting in vision impairment. Hence, we recommend the early referral of such patients to ophthalmology, even with seemingly mild ocular symptoms.

## Conclusions

Our case illustrates that viral keratitis can present in post-renal transplant recipients on immunosuppression with minimal symptoms but florid signs (dendritic ulcers near the limbus and multiple dendrites) without prior herpetic eye or skin/mucosal disease. However, it can be successfully treated with the currently available antiviral therapy of topical ganciclovir or acyclovir (supplemented by oral or systemic acyclovir or valacyclovir in stromal keratitis or keratouveitis). Hence, early ophthalmology referral with a high index of suspicion and the institution of appropriate therapy may be crucial in preventing vision-impairing sequelae.
